# Endothelial anthrax toxin receptor 2 plays a protective role in liver fibrosis

**DOI:** 10.3389/fcell.2023.1278968

**Published:** 2024-01-23

**Authors:** Xiaojuan Huang, Liyin Zhang, Wei Luo, Yu Zeng, Xiaoxue Li, Nan Yang, Wenwen Huang, Bi-Sen Ding

**Affiliations:** Key Laboratory of Birth Defects and Related Diseases of Women and Children of MOE, State Key Laboratory of Biotherapy, West China Second University Hospital, Sichuan University, Chengdu, China

**Keywords:** ANTXR2, endothelial cells, liver fibrosis, MMP2, extracellular matrix

## Abstract

Hepatocellular carcinoma is one of the leading cancers worldwide and is a potential consequence of fibrosis. Therefore, the identification of key cellular and molecular mechanisms involved in liver fibrosis is an important goal for the development of new strategies to control liver-related diseases. Here, single-cell RNA sequencing data (GSE136103 and GES181483) of clinical liver non-parenchymal cells were analyzed to identify cellular and molecular mechanisms of liver fibrosis. The proportion of endothelial subpopulations in cirrhotic livers was significantly higher than that in healthy livers. Gene ontology and gene set enrichment analysis of differentially expressed genes in the endothelial subgroups revealed that extracellular matrix (ECM)-related pathways were significantly enriched. Since anthrax toxin receptor 2 (ANTXR2) interacts with the ECM, the expression of ANTXR2 in the liver endothelium was analyzed. ANTXR2 expression in the liver endothelium of wild-type (WT) mice significantly decreased after a 4-time sequential injection of carbon tetrachloride (CCl_4_) to induce liver fibrosis. Next, conditional knockout mice selectively lacking *Antxr2* in endothelial cells were generated. After endothelial-specific *Antxr2* knockout mice were subjected to the CCl_4_ model, the degree of liver fibrosis in the knockout group was significantly more severe than that in the control group. In addition, ANTXR2 in human umbilical vein endothelial cells promoted matrix metalloproteinase 2 (MMP2) activation to degrade the ECM *in vitro*. Finally, endothelial-specific overexpression of *Antxr2* alleviated the development of liver fibrosis following adeno-associated virus treatment. Collectively, these results suggested that endothelial ANTXR2 plays a protective role in liver fibrosis. This function of ANTXR2 may be achieved by promoting MMP2 activation to degrade the ECM.

## 1 Introduction

Due to lifestyle changes, the burden of hepatocellular carcinoma (HCC) has shifted from viral causes of chronic injury, such as hepatitis B and C, to non-viral causes of chronic injury, such as alcohol (Huang et al., 2023) or metabolic syndrome inducing non-alcoholic steatohepatitis (Friedman et al., 2018), and reflects the rapid increase in the incidence of liver cancer (Toh et al., 2023). Liver fibrosis, a fibrous scar that develops with the accumulation of extracellular matrix (ECM) proteins replacing damaged normal tissue, is a poor wound healing response of the liver to multiple causes of chronic injury (Pellicoro et al., 2014). Liver cirrhosis, and eventually liver cancer, occurs when persistent damage and excessive scarring lead to changes in tissue function (Bataller and Brenner, 2005; Schuppan et al., 2018b). Although several classes of drugs have been developed to treat liver fibrosis of different etiologies, stages, and progressions (Kisseleva and Brenner, 2021), there are currently no approved treatments. Thus, the identification of the mechanisms involved in liver fibrosis is an important goal for the development of new strategies to control liver-related diseases.

Liver sinusoidal endothelial cells (LSECs), which represent the interface between blood cells on one side and hepatocytes and hepatic stellate cells on the other (Poisson et al., 2017), are among the most abundant non-parenchymal hepatic cell populations. In addition to forming a barrier within the hepatic sinusoid, the luminal surface of LSECs serves as a platform for interactions among membrane proteins (Shetty et al., 2018). After organ injury, the spatial and temporal coordination of vascular factors in the tissue-specific endothelium plays a crucial role in adaptive healing and fibrotic remodeling (Rafii et al., 2016; Gracia-Sancho et al., 2021). This inherent proregenerative potential of tissue-specific endothelial cells (ECs) might be exploited therapeutically to coordinate fibrosis-free healing and restore homeostasis in tissues.

Anthrax toxin receptor 2 (ANTXR2), also known as capillary morphogenesis gene 2, was first reported to be differentially expressed during capillary morphogenesis in three-dimensional collagen matrices (Bell et al., 2001). ANTXR2 is a type I transmembrane protein that functions as an anthrax toxin receptor (Sun and Pedro, 2016) and has broad ligand specificity for ECM proteins (Cryan et al., 2022). Female *Antxr2* ^−/−^ mice suffer from severe progressive fibrosis of the uterus, leading to structural disruption and delivery failure (C. V. Reeves et al., 2012), (C. V. Reeves et al., 2010). Loss of ANTXR2 function promotes collagen VI accumulation in patients, leading to nodule formation (Bürgi et al., 2017). Liver fibrosis also exhibits excessive deposition of ECM in the dissection space between ECs and adjacent cells (Bataller and Brenner, 2005). Therefore, we speculated that ANTXR2 also plays an important role in the liver fibrosis process.

In this study, the mechanisms of liver fibrosis were explored and we report that a novel function of ANTXR2 in the liver and a potential therapeutic target for liver fibrosis. ANTXR2 regulated the balance between liver repair and fibrosis. This may be because the expression of ANTXR2 in ECs promotes the activation of matrix metalloproteinase 2 (MMP2) to degrade the ECM. Taken together, these data suggested that ANTXR2 is the key factor regulating the activity of MMP2 in ECs, and that endothelial ANTXR2 plays a protective function in liver fibrosis.

## 2 Materials and methods

### 2.1 Mice

C57BL/6J mice were obtained from Shanghai Model Organisms Co., Ltd. (Shanghai, China). *Antxr2* ^loxP/loxP^ mice were obtained from GemPharmatech Co., Ltd. (Nanjing, China). EC-specific Cdh5-(PAC)-Cre^ERT2^ were provided by Dr. Ralf Adams (Y. Wang et al., 2010). *Antxr2* ^loxP/loxP^ mice were crossed with Cdh5-Cre^ERT2^ mice carrying tamoxifen response-Cre driven by an EC-specific Cdh5/vascular endothelial (VE)-cadherin promoter to establish VE-cadherin-Cre^ERT2^ *Antxr2* ^loxP/loxP^ mice. To induce EC-specific genetic deletion of *Antxr2* (*Antxr2* ^iΔEC/iΔEC^), at approximately 6–8 weeks of age *Antxr2* ^loxP/loxP^ mice and VE-cadherin-Cre^ERT2^ *Antxr2* ^loxP/loxP^ mice were intraperitoneally treated with tamoxifen at a dose of 150 mg/kg for 6 days interrupted for 3 days after the third dose (1g tamoxifen was diluted in 50 mL corn oil to yield a concentration of 20 mg/mL). The deletion of target genes in liver ECs was corroborated using quantitative polymerase chain reaction (qPCR) analysis on the 14th day after the last injection. Genotyping and qPCR primers were shown in Table 1 of the Supplementary Material. The animal study was approved by the Institutional Animal Care and Use Committee of West China Second University Hospital (protocol code2019026, date of approval 1 March 2019).

### 2.2 Cell lines

Human umbilical vein endothelial cells (HUVECs) were cultured in EndoGRO-VEGF Complete Culture Media (S. Rafii et al., 1994) (SCME 002-5, Millipore). HEK293T cells were cultured in DMEM full medium (C11995500, Gibico) with 10% FBS at 37°C in a humidified atmosphere of 5% CO_2_.

### 2.3 Data source and preprocessing

The scRNA seq datasets of GSE136103 and GSE181483 were downloaded from the GEO database, which included 5 cirrhotic human liver samples and 6 healthy human liver samples. The data contained a total of 23,737 genes and 71,164 cells. The percentage of mitochondria and rRNA was calculated through the Percentage Feature Set function, and the genes expressed by each cell were greater than 200 and less than 5000.

### 2.4 scRNA-seq data clustering dimension reduction

#### 2.4.1 Data normalization

Data was normalized by the “SCTransform” function, which is a normalization method for single-cell UMI count data using a variance stabilizing transformation, in sctransform R package. The transformation is based on a negative binomial regression model with regularized parameters. As a part of the same regression framework, this package also provides functions for batch correction, and data correction.

#### 2.4.2 Batch effect remove

Batch effects among the samples were observed and corrected. Function “RunHarmony” in Harmony R package was utilized to remove batch effect among different samples.

#### 2.4.3 Clustering and visualization

“RunPCA” was used to select top 50 principal components for further dimension reduction of PCA for the SCT data. “FindNeighbors” and “FindClusters” functions were adopted to identify cell clusters (resolution = 0.1) and the clusters were visualized with Uniform Manifold Approximation and Projection (UMAP). Mainly based on the theory of manifold theory and topology algorithm, the dimensionality of high-dimensional data was reduced to form the input features of other classification models. All the functions were built-in functions of Seurat R package.

#### 2.4.4 Markers identification

“FindAllMarkers” function helped recognize marker genes of 9 subgroups and cell markers used in published articles were referred as well. Graph-based clustering of cells identified nine subclusters with uniform manifold approximation and projection signature genes in each cluster, cross-referenced with known marker genes of the cell population, and annotated the different cell types.

### 2.5 Mouse liver fibrosis models

Injection of carbon tetrachloride (CCl_4_) was used to induce liver injury as previously described (Mengli et al., 2023; Qing et al., 2022; Zhou et al., 2022). CCl_4_ (319961, Sigma-Aldrich) was diluted in corn oil (c8267, Sigma-Aldrich) to yield a concentration of 40% (0.64 mg/mL). 6–8 weeks mice were intraperitoneally injected with 40% CCl_4_ every 3 days for four consecutive injections, with samples harvested on the second day after the last injection. The WT-Vehicle group was intraperitoneally injected with oil.

### 2.6 Sirius red staining

Collected tissues were fixed in 4% paraformaldehyde, embedded in paraffin and sectioned into 6 μm. Sections were heated in 65°C drying oven for 1 h, xylene deparaffinized and then rehydrated with graded ethanol. Then the sections were gently washed by water and dried. After that, the sections were stained with Sirius red dye (BP-DL030, Solarbio) for 15 min and gently rinsed with running water. Finally, sections were dehydrated using graded ethanol, transparented by xylene and sealed with resinene.

### 2.7 Hematoxylin and eosin staining

Collected tissues were fixed in 4% paraformaldehyde, embedded in paraffin and sectioned into 6 μm. Sections were heated in 65°C drying oven for 1 h, xylene deparaffinized and then rehydrated with graded ethanol. Then nucleus was stained with Hematoxylin (G1120, Solarbio) for 1 min and rinsed in running water for 10 min. After that liver sections were differentiated with 75% alcohol containing 1% hydrochloric acid buffer for 1 min and washed for 30 s with running water. Then sections were re-dyed with Eosin Y Aqueous Solution (G1120, Solarbio) for 2 min. Finally, sections were dehydrated using graded ethanol each for 2 s, transparented by xylene and sealed with resinene.

### 2.8 Hydroxyproline assay

Approximately 0.2 g of the liver sample was cut off as finely as possible in 5 mL tube and 2 mL 6 mol/L hydrochloric acid was added to tube. Then the extraction solution was boiled at 110°C for 4 h until no visible large lumps. After cooling at room temperature, the pH value of the extraction solution should be adjusted to within the range of 6–8 using 10 mol/L NaOH, and the total volume of the mixture should be diluted to 4 mL with distilled water. Finally, the mixture was centrifuged at 13000 g for 20 min. The supernatant was used for the determination of hydroxyproline. Hydroxyproline content was carried out followed by protocol of Hydroxyproline Colorimetric Assay Kit (BC0255, Solarbio). The OD value of each well was measured at 560 nm with a SpectraMax Absorbance CMax PLUS reader.

### 2.9 Blood biochemistry assay

Blood samples were collected and stored overnight at 4°C. Samples were centrifuged at 3,000 *g* for 30 min at 4°C, and the supernatant was serum. The serum levels of alanine transaminase (ALT), aspartate transaminase (AST), and ALP (alkaline phosphatase) were measured by the automated biochemical analyzer (Roche, Beijing, China).

### 2.10 Isolation of primary hepatocytes and ECs

The liver tissues were mechanically broken, digested with 10 mL digestion solution which was made up with PBS containing collagenase I (17100017, Invitrogen) at a concentration of 2 mg/mL. The digestion solution containing the liver tissue was spun at 30 rpm for 40 min in a 37°C incubator. The digestion solution was pipetted 5 times with a 18G blunt syringe, and filtered with a 40 μm cell filter. The filtrate was centrifuged at 300 g for 3 min. The bottom cells were resuspended with 10 mL of red blood cell lysis solution and lysed on ice for 10 min to remove red blood cells. Cells were washed once with PBS and centrifuged at 50 g for 5 min. The bottom cells were hepatocytes (Cao et al., 2017). The supernatant was centrifuged at 300 g for 3 min again and the obtained precipitation was non-parenchymal cells (NPCs). In order to obtain endothelial cells, the magnetic beads (319961, Invitrogen) were incubated with CD45 antibody (553076, BD), or CD31 antibody (553370, BD) for 4 h in advance. Then CD45 antibody-coated beads were incubated with NPCs at 4°C for 40 min, and the CD45-positive cells were adsorbed on a magnetic stand. The Magnetic-Activated Cell Sorting (MACS) buffer was used when cells were incubated with antibody-coated beads. The MACS buffer was PBS containing 0.5% BSA and 2 mmol/L EDTA. The CD45-negative cells were incubated with CD31 antibody-coated beads at 4°C for 40 min, and the CD31-positive cells were adsorbed on a magnetic stand. CD45^−^CD31^+^ endothelial cells were finally obtained after washing 6 times with MACS buffer.

### 2.11 Zymography assay

The activity of MMP2 secreted by HUVECs was detected with Zymography assay kit (XF-P17750, GEMIC). The same amount of HUVECs was seeded into a six-well plate and cultured for 24 h. The complete medium then was replaced with fresh serum-free culture medium and HUVECs were cultured for another17 h. The supernatant was collected from the cell culture medium by centrifuging at 300 g for 3 min at 4°C. Simultaneously, HUVECs were digested with trypsin and counted to correct the sample loading amount during zymogram detection. The supernatant was transferred to a 3 kDa ultrafiltration tube (UFC500308, Millipore) and centrifuged at 4,000 *g* for 60 min at 4°C. Non-reducing SDS loading buffer was added to the supernatant and then samples were subjected to electrophoresis analysis. The gel was washed with a shaking incubator using a 2.5% Triton-X100 solution for 1 h, then replaced with a fresh 2.5% Triton-X100 solution and incubated at 37°C for 1 h. This step was repeated once. The gel was incubated in buffer A solution from Zymography assay kit at 37°C for 5 h. Then the gel was incubated in buffer B solution from Zymography assay kit at 37°C for 12 h. The gel was stained with Coomassie brilliant blue staining solution on a horizontal shaker for 2–3 h. The stained gel was rinsed with destaining solution, which was composed of 7% glacial acetic acid, 5% methanol and 88% distilled water. Most areas of the gel would be stained, except for the bands near the active MMP2 and MMP9 bands. The gel was destained until clear enzymogram bands appeared on a clear background with no color. The images of the gels were captured and recorded with Gel Doc™ EZ Imager (Bio-Rad, CA, United States).

### 2.12 Immunofluorescence

Frozen slide of liver tissue after 40% CCl_4_-induced fibrosis modeling and hepatocytes slide were washed 3 times with PBS on a shaker for 5 min. The slides were fixed with 4% PFA for 10 min and washed 3 times with PBS, then permeabilized with blocking solution for 10 min. The blocking solution was PBS containing 1% donkey serum and 0.3% Triton-X100. Then the slides were incubated with primary antibodies: CD31 (GB13063, Servicebio), α-SMA (14395-1-AP, Preoteintech), Collagen I (ab34710, Abcam), ANTXR2 (PA5-76013, Invitrogen) overnight at 4°C and the dilution ratio was 1:100. The antibody dilution buffer was PBS containing 0.3% Triton-X100. On the second day slides were washed with PBS three times. Then slides were incubated with secondary antibody including Alexa Fluor^®^ 647 (715-605-150, Jackson ImmunoResearch), Alexa Fluor^®^ 555 (ab150074, Abcam) at a dilution ratio of 1:200 for 1 h at 37°C in an incubator. The nuclei were stained with DAPI (D3571, Thermo Fisher Scientific) for 5 min. And the slides mounted with a cover glass. Fluorescent images were recorded on AxioVert LSM 980 confocal microscope (ZEISS), and processed using ZEN (Zeiss).

### 2.13 Plasmid construction and recombinant protein expression

shRNA recombinant lentiviruses targeting human *ANTXR2* were constructed on the basis of the pGLVU6/Puro vector. The LV2 plasmids containing shRNA sequences and the helper plasmids (pMD2.G and pSPAX2) encoding the skeleton structure were co-transfected into the HEK293T (Qing et al., 2022). To construct constitutively overexpression *ANTXR2* (OE-ANTXR2), the human *ANTXR2* open reading frame was amplified using HUVEC cDNA and cloned into plenti-C-GFP vector from our lab. This vector was used to construct an overexpressing virus and infect cells in the same way as above. Sequences of the primers used for plasmid construction were listed in Table 1 of the Supplementary Material. All of the knockdown and overexpression cell lines were confirmed by western blot and quantitative polymerase chain reaction (qPCR) analysis.

### 2.14 Endothelium specific overexpression *Antxr2*

The custom-made Adeno-associated virus with full-length cDNA of mouse *Antxr2* was purchased from Hanbio technology Co. Ltd. AAV-NC virus was also provided by the company as controls. These Adeno-associated virus were injected into mice to overexpress *Antxr2*. In brief, mice were subjected to a 2-cm long incision on the right side of the abdomen near the thorax under general anesthesia. 100 μL of AAV (1 × 10^12^ v.g/mL) was slowly injected into the exposed spleen. After the injection was completed, the wound was sutured up after 10 minutes of gentle pressure on the bleeding site with a hemostatic cotton.

### 2.15 Western blot analysis

HUVECs and liver tissues were lysed in RIPA buffer (20-188, Millipore) containing a protease inhibitor cocktail (11088793001, Roche) and a phosphatase inhibitor cocktail (11088793001, MCE). Cell lysate was centrifuged at 13,000 g for 30 min at 4°C and the supernatant was retained. The obtained samples were detected by immunoblotting experiments. The immunoblots were incubated with the blocking buffer which was 0.2% TBS’T buffer with 5% BSA. And the immunoblots were incubated with the primary antibodies against beta-Actin (GB12001, servicebio), Collagen I (ab34710, Abcam), α-SMA (ab34710, Abcam), ANTXR2 (PA5-76013, Invitrogen) at a dilution of 1:1000 for 8 h at 4°C. The immunoblots were washed 3 times using TBS’T buffer. Next, the immunoblots were incubated with secondary antibody at room temperature for 2 h and washed 3 times with TBS’T buffer. The corresponding secondary antibodies were HRP-sheep-anti-mouse (GB23301, Servicebio) and HRP-sheep-anti-rabbit (GB23303, Servicebio) at a dilution of 1:3,000. Western blot images were captured with Touch Imager XLi (E-BLOT, shanghai, China). These experiments were performed as previously described (Qing et al., 2022).

### 2.16 Gene expression analysis

Total RNA was extracted from harvested cells or frozen liver tissues using TRIzol™ (15596026, Thermo Fisher Scientific), following Trizol’s protocol. Then 500 ng of total RNA was reversed transcription into cDNA by the PrimeScript™ RT reagent Kit (RR047A-1, Takara), following Kit’s protocol. The cDNA expression of specific genes was detected using SYBR Green Master Mix (Q712-02/03, Vazyme) on a CFX96 Real-Time PCR system (Bio-Rad, CA, United States). Gene expression analysis was performed as previously described (Qing et al., 2022). qPCR primers were shown in Table 1 of the Supplementary Material.

### 2.17 Flow cytometry analysis

1 × 10^6^ cells were preblocked with PBS containing 1% donkey serum for half an hour. Single cell suspensions were incubated with CD31 and CD45 antibodies (Human:555482, BD; 555446, BD; Mouse:550994, BD; 553373, BD) at 4°C for 30 min and the dilution ratio was 1:200. Labeled cell populations were measured by a Fortessa flow cytometer (Beckton Dickenson, NY, United States). Flow cytometry analysis was performed using unstained control samples for determining appropriate gates, voltages. HUVECs phenotypes with CD45^−^CD31^+^. The results were analyzed with Flow Jo V10.

### 2.18 Image acquisition and analysis

Sirius red, and HE staining of sections were captured with 3D Panoramic MIDI (3D Panoramic MIDI, Budapest, Hungary). Densitometry analysis of immunoblot images and Sirius red positive area was carried out using ImageJ software. Figures 3A, 4B, 6A and Figure 8 were drawn by Figdraw.

### 2.19 Quantification and statistical analysis

Calculations were carried out with GraphPad Prism 8 (GraphPad Software, CA, United States). Statistical analysis of difference between more than two groups were performed with one-way ANOVA and Tukey’s test as *post hoc* analysis. Comparisions between two groups, Student’s t-test was employed to determine significant differences. *p*-value of less than 0.05 was considered statistically different and expressed as *, *p* < 0.05, **, *p* < 0.01; ***, *p* < 0.001and ****, *p* < 0.0001. All results are shown as mean ± S.D.

## 3 Results

### 3.1 The endothelial ratio of non-parenchymal cells in fibrotic liver increased

**FIGURE 1:**
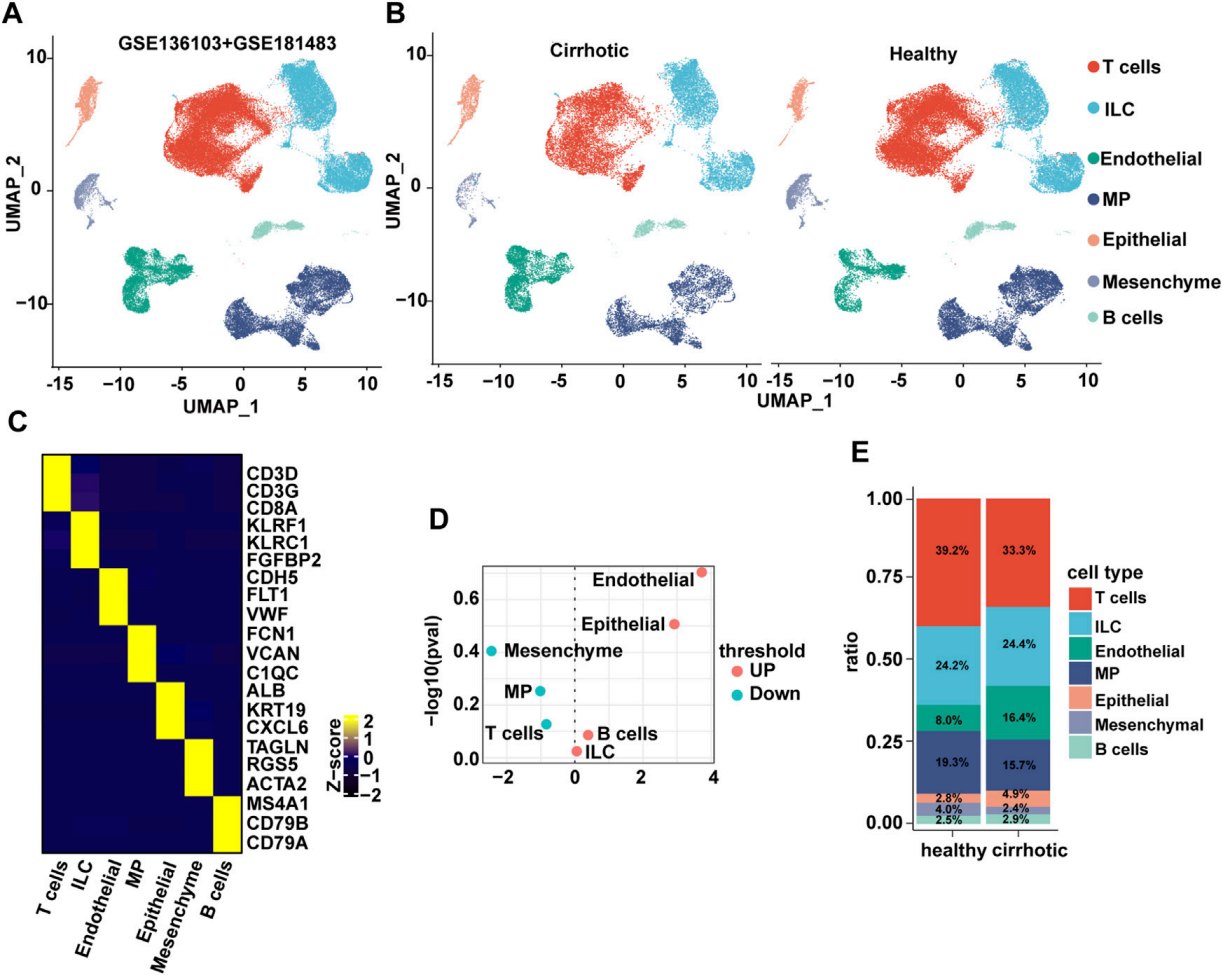
The endothelial ratio of non-parenchymal cells (NPCs) in fibrotic liver is increased. **(A)** Clustering of 71,164 cells from 6 healthy and 5 cirrhotic human livers. **(B)** Cell types are annotated and split into two groups. Cell lineage is inferred from expression of marker gene signatures. ILC, innate lymphoid cell; MP, mononuclear phagocyte. **(C)** Heatmap of cluster marker genes, exemplar genes (right), and lineage annotation (bottom). Columns represent cells and rows represent genes. **(D)** Volcano plot depicting differences in cell type abundance in the cirrhotic group compared to that of the healthy group. Fold change (log2) is plotted against *p*-value (−log10) based on a Student’s t-test. **(E)** Distribution of cell types in human liver samples from healthy and cirrhotic groups.

The liver is composed of parenchymal cells (i.e., mainly hepatocytes) and non-parenchymal cells (NPCs) such as hepatic stellate cells, endothelial cells, and immune cells (H. Zhang et al., 2021). The single-cell RNA sequencing (scRNA seq) datasets (GSE136103 and GSE181483) from the Gene Expression Omnibus database were analyzed, which allowed the dissection of the cellular and molecular basis of liver fibrosis at the single-cell level (Ramachandran et al., 2019; H; Zhang et al., 2021). After filtering, 71,164 cells comprising 28,627 cells from patients with cirrhosis and 42,537 cells from healthy controls were retained. Nine major cell types were characterized: B cells, ECs, innate lymphoid cells, mononuclear phagocyte, epithelial cells, mesenchymal cells, and T cells (Figures 1A–C). Surprisingly, when a *t*-test was conducted on the cell type proportion of samples, the proportion of ECs changed the most and increased significantly in the cirrhotic group (Figures 1D,E).

### 3.2 Decreased expression of *ANTXR2* in human cirrhotic liver ECs

**FIGURE 2:**
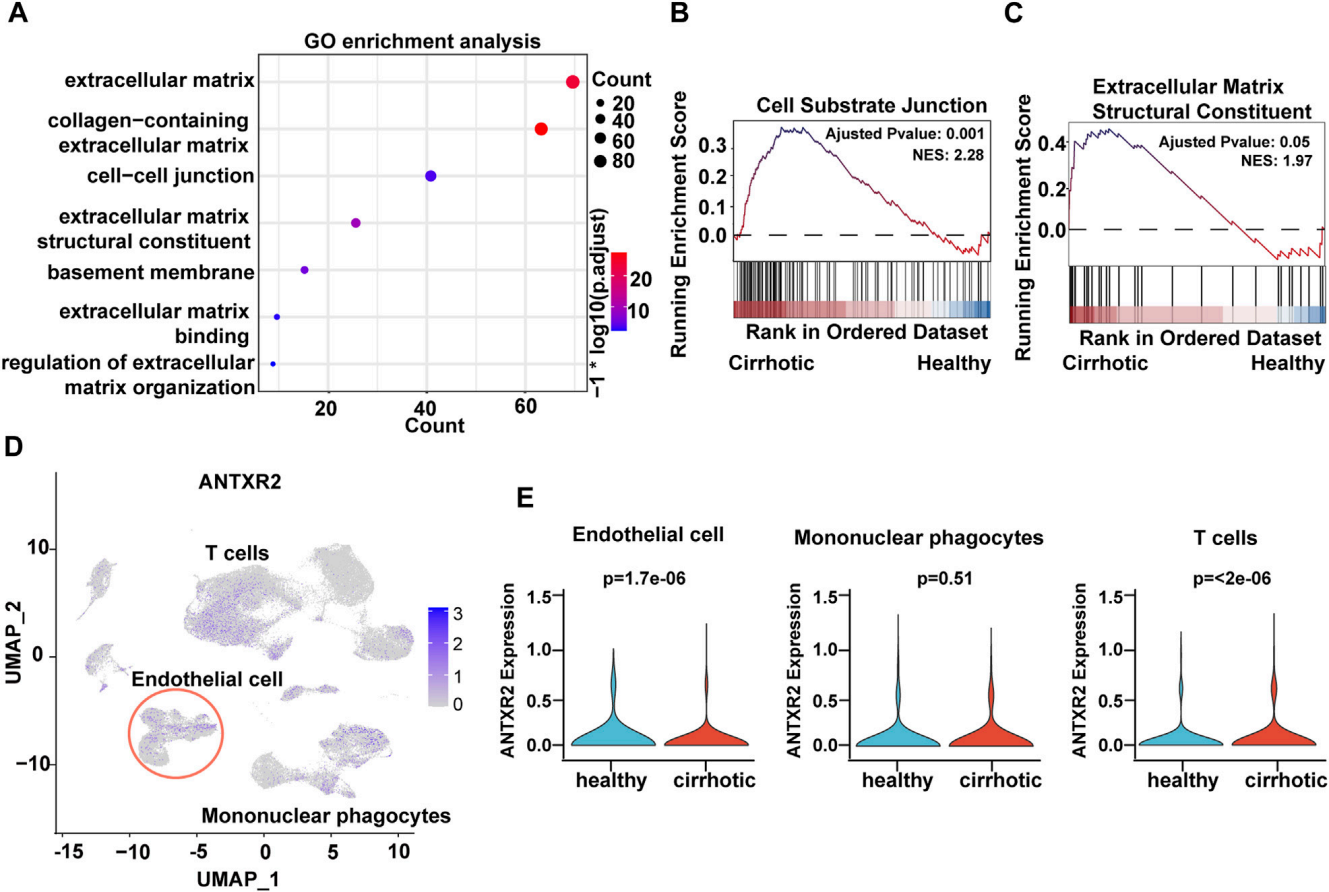
Decreased expression of anthrax toxin receptor 2 (Antxr2) in human cirrhotic liver endothelial cells. **(A)** Gene ontology (GO) enrichment analysis of differentially expressed genes (DEGs) between cirrhotic and healthy endothelial cells. **(B, C)** Gene set enrichment analysis (GSEA) analysis of DEGs in cirrhotic and healthy endothelial cells. The basement membrane and extracellular matrix structural constituent pathways are shown. **(D)** UMAP visualization of ANTXR2 expression in non-parenchymal cells. **(E)** Violin plot showed ANTXR2 expression in endothelial cells, mononuclear phagocytes and T cells from healthy and cirrhotic human livers.

To investigate the expression features of cirrhotic ECs, the FindMarkers function with default parameters (log_2_fc threshold = ± 0.25) was used to identify differentially expressed genes (DEGs) between the two groups (cirrhotic and healthy ECs). Next, gene ontology (GO) and gene set enrichment analysis (GSEA) of the DEGs were performed. Multiple pathways related to the ECM were enriched and GSEA showed that the cell-substrate junction and ECM structural constituent pathways were activated in cirrhotic ECs (Figures 2A–C). ANTXR2 is a transmembrane protein that interacts with the ECM to connect cells and substrates (Sun and Pedro, 2016). Recent research has shown that uterus-verified ANTXR2 knockout leads to severe fibrosis (16). Liver fibrosis also results in excessive deposition of ECM (Bataller and Brenner, 2005; Asrani et al., 2019); therefore, whether liver endothelial ANTXR2 plays an important role in the progression of liver fibrosis was investigated. The uniform manifold approximation and projection results showed that Antxr2 expressed in endothelial cells, mononuclear phagocyte and T cells (Figure 2D). Compared with the healthy group, the expression of *ANTXR2* was downregulated in endothelial cells and upregulated in T cells in the cirrhosis group, but had no change in mononuclear phagocytes (Figure 2E).

### 3.3 Decreased expression of *Antxr2* in mice fibrotic liver ECs

**FIGURE 3:**
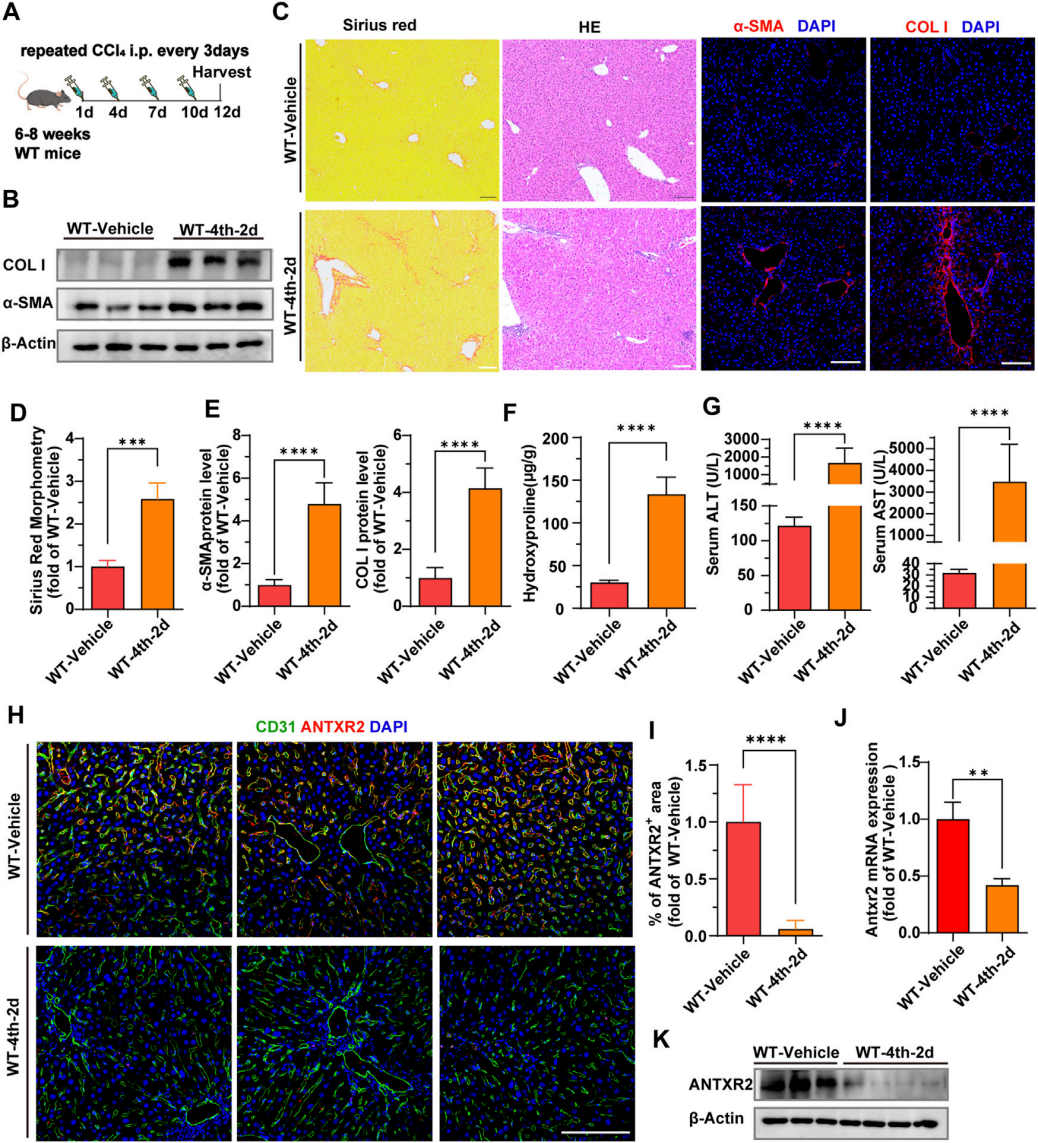
Decreased expression of anthrax toxin receptor 2 (Antxr2) in mice fibrosis liver endothelial cells. **(A)** Sequential injection of carbon tetrachloride (CCl_4_) to induce chronic liver injury in wild-type mice (WT). WT mice aged 6–8 weeks were intraperitoneally injected with 40% CCl_4_ every 3 days for 4-time consecutive injection, with samples harvested on the second day after the last injection (WT-4th-2d). The WT-vehicle group was intraperitoneally injected with oil (*n* = 6 animals per group). **(B–E)** Fibrotic responses in WT mice after fibrosis modeling. Levels of α-smooth muscle actin (α-SMA) and collagen I **(B,C, E)**, Sirius red staining **(C, D)**, and hematoxylin and eosin **(H, E)** staining **(C)** are measured to assess the degree of liver fibrosis (*n* = 6 animals per group). Scale bar = 100 μm. **(F)** Amounts of hydroxyproline in indicated mice (*n* = 5–10 animals per group). **(G)** Levels of serum alanine aminotransferase (ALT) and aspartate aminotransferase (AST) in WT-4th-2d group and WT-Vehicle group mice. (n = 9–10 animals per group). **(H)** Co-staining of endothelial marker CD31 (green) and ANTXR2 (red) in the liver of indicated mice groups. Scale bar = 100 μm. **(I)** Quantification of the area of the ANTXR2^+^ region in indicated mice. (*n* = 7 animals per group). **(J, K)** *Antxr2* mRNA and protein expression in liver endothelial cells (ECs, CD45^−^CD31^+^) from WT mice after fibrosis modeling (*n* = 3–6 animals per group). Student’s t-test was employed to determine significant differences. The results of all bar graphs are expressed as mean ± S.D. *p* < 0.01**, *p* < 0.001***; *p* < 0.0001****.

In order to further explore the level and distribution of Antxr2 expression in mouse fibrotic liver, chronic liver fibrosis was induced in wild-type (WT) mice by four consecutive injections of carbon tetrachloride (CCl_4_) (WT-4th-2d) (Figure 3A). Western blot, Sirius red staining, immunofluorescence staining, and qPCR results showed that the expression levels of fibrosis-related molecules, such as α-smooth muscle actin (α-SMA) and collagen I were significantly increased in the WT-4th-2d group mice compared to that of the WT-vehicle control group (Figures 3B–E). Hydroxyproline is an amino acid predominantly found in collagen, and its quantity is positively correlated with the degree of fibrosis (Song et al., 2022). The hydroxyproline content in mice in the WT-4th-2d group was significantly higher than that of mice in the WT-vehicle group (Figure 3F). Alanine aminotransferase (ALT) and aspartate aminotransferase (AST), which are important indicators of liver function, increased significantly in the WT-4th-2d group compared to that of the control group (Figure 3G). These data indicated that four consecutive injections of CCl_4_ induced chronic liver fibrosis in WT mice. Since the human liver NPCs scRNA seq data analysis showed that the proportion of ECs in the cirrhosis group changed the most and extracellular matrix-related pathways were enriched in endothelial cell differential genes, endothelial markers CD31 and ANTXR2 were first co-stained in the liver. ANTXR2 was mainly expressed in mouse liver ECs (Figure 3H). And its expression was downregulated after CCl_4_ injection in WT mice (Figures 3H,I). Next, liver ECs of WT mice after CCl_4_ modeling were sorted using magnetic beads to analyze *Antxr2* expression. Excitingly, *Antxr2* expression in liver ECs of mice in the WT-4th-2d group was significantly lower than that in the control group (Figures 3J,K, Supplementary Figure S1). The above results are consistent with reduced endothelial ANTXR2 expression in human cirrhotic liver. Vascular microenvironment plays critical role in liver fibrosis and regeneration (Rafii et al., 2016). Therefore, we focused on the effect of endothelial ANTXR2 expression on liver fibrosis.

### 3.4 Liver fibrosis increased in endothelium-specific *Antxr2* knockout mice after fibrosis modeling

**FIGURE 4:**
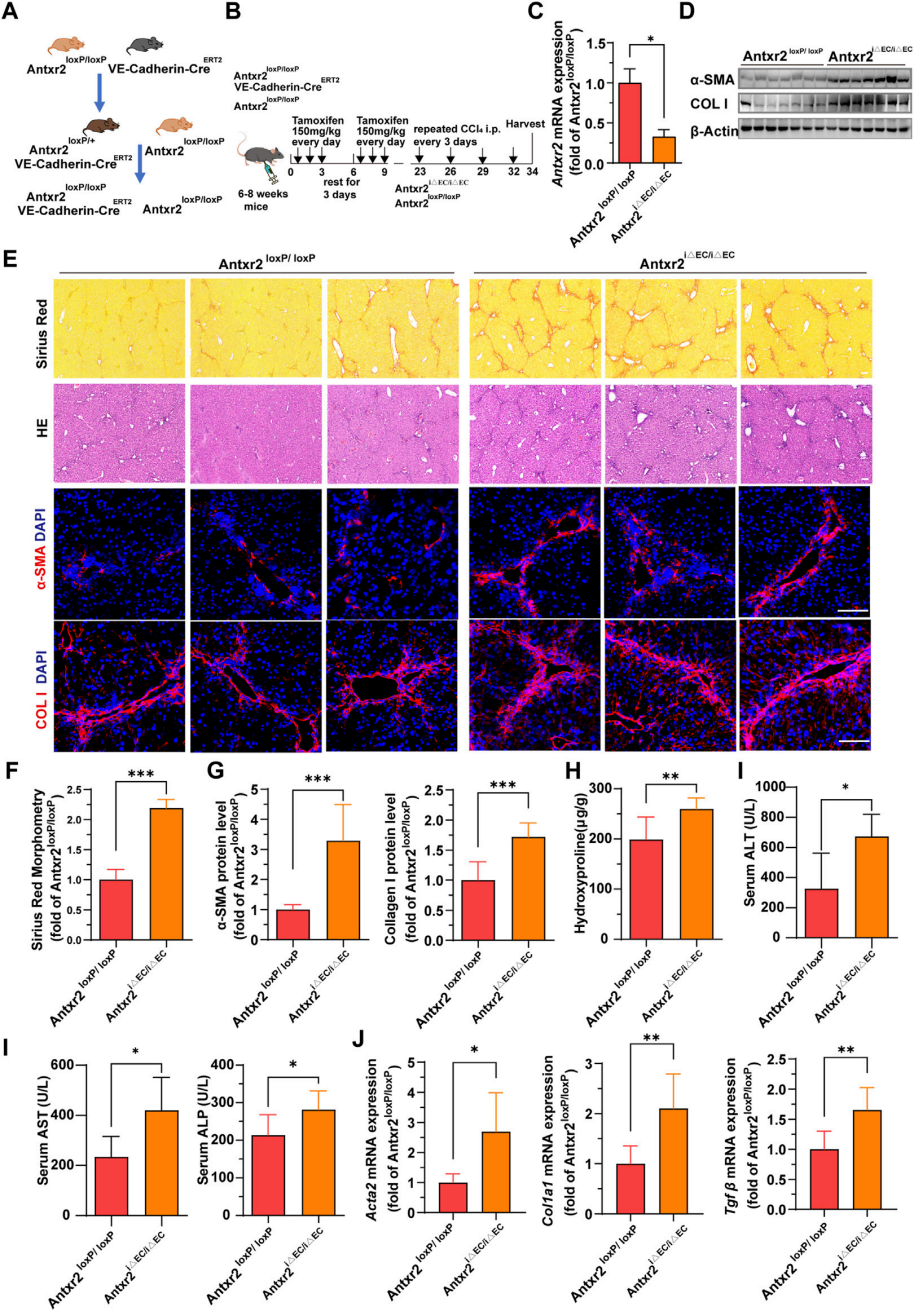
Liver fibrosis increases in endothelium-specific anthrax toxin receptor 2 (*Antxr2*) knockout mice after fibrosis modeling. **(A, B)** Generation of inducible endothelial cell (EC)-specific deletion of *Antxr2* in adult mice. Floxed *Antxr2* (*Antxr2* ^loxP/loxP^) mice are crossed with Cdh5-Cre^ERT2^ mice carrying tamoxifen response-Cre driven by an EC-specific Cdh5/vascular endothelial (VE)-cadherin promoter. To induce EC-specific genetic deletion of *Antxr2*, at approximately 6–8 weeks of age *Antxr2* ^loxP/loxP^ mice and VE-cadherin-Cre^ERT2^ *Antxr2* ^loxP/loxP^ mice are intraperitoneally treated with tamoxifen at a dose of 150 mg/kg for 6 days interrupted for 3 days after the third dose. Then, *Antxr2* ^iΔEC/iΔEC^ mice and age-matched littermate mice undergo carbon tetrachloride (CCl_4_) liver injury modeling. **(C)** Inducible knockout efficiency of *Antxr2* in liver ECs of *Antxr2* ^iΔEC/iΔEC^ mice is quantified in sorted CD45^−^CD31^+^ ECs using quantitative polymerase chain reaction (qPCR) (*n* = 3 animals per group). **(D–G)** Fibrotic responses in *Antxr2* ^iΔEC/iΔEC^ and *Antxr2* ^loxP/loxP^ mice after fibrosis modeling. Levels of α-smooth muscle actin (α-SMA) and collagen I **(D, E, G)**, Sirius red staining **(E)**, quantification of Sirius red staining **(F)** and hematoxylin and eosin (HE) staining **(E)** are measured to assess the degree of liver fibrosis (*n* = 7 animals per group). Scale bar = 100 μm. **(H)** Amounts of hydroxyproline in *Antxr2* ^iΔEC/iΔEC^ and *Antxr2* ^loxP/loxP^ mice after fibrosis modeling (*n* = 5–6 animals per group). **(I)** Levels of serum alanine aminotransferase (ALT), aspartate aminotransferase (AST), and alkaline phosphatase (ALP) in indicated mice (*n* = 5–6 animals per group). **(J)** mRNA expression levels of fibrosis-related genes in *Antxr2* ^iΔEC/iΔEC^ and *Antxr2* ^loxP/loxP^ mice after fibrosis modeling. (*n* = 6 animals per group). Student’s t-test was employed to determine significant differences. The results of all bar graphs are expressed as mean ± S.D. *p* < 0.05*; *p* < 0.01**, *p* < 0.001***.

We hypothesized that ANTXR2 serves as a bridge connecting ECs and the ECM, thereby influencing the occurrence and progression of liver fibrosis. To test this hypothesis, endothelial-specific *Antxr2* knockout mice were generated and the functional influence of endothelial ANTXR2 on liver repair was explored. EC-specific knockout of *Antxr2* mice was generated by breeding floxed *Antxr2* (*Antxr2* ^loxP/loxP^) mice with mice carrying tamoxifen-responsive Cre^ERT2^recombinase driven by EC-specific vascular endothelial (VE)-cadherin/Cdh5 promoter (Cdh5-(PAC)-Cre^ERT2^) (Figure 4A). To induce EC-specific genetic deletion of *Antxr2*, at approximately 6–8 weeks of age *Antxr2* ^loxP/loxP^ mice and VE-cadherin-Cre^ERT2^ *Antxr2* ^loxP/loxP^ mice were intraperitoneally treated with tamoxifen at a dose of 150 mg/kg for 6 days, interrupted for 3 days after the third dose. After 2 weeks of tamoxifen treatment (Figure 4B), the deletion of target genes in liver ECs was corroborated using quantitative PCR analysis. The knockout efficiency of Antxr2 in liver ECs was approximately 70% in Antxr2 ^iΔEC/iΔEC^ mice, but not in hepatocytes and NPCs except endothelial cells (Figure 4C, Supplementary Figure S2A, B). *Antxr2* ^iΔEC/iΔEC^ mice were subjected to four consecutive injections of CCl_4_ to induce liver fibrosis (Figure 4B). Tamoxifen-treated *Antxr2* ^loxP/loxP^ *Cre* ^*-*^ mice (age/sex/weight matched littermate mice) were utilized as controls (*Antxr2* ^loxP/loxP^). The expression level of fibrotic markers, such as α-SMA and collagen I, was measured via immunoblotting, immunofluorescence staining, and qPCR analysis. The expression of α-SMA and collagen I was significantly increased in the *Antxr2* ^iΔEC/iΔE*C*^ mice compared to that of the *Antxr2* ^loxP/loxP^ mice (Figures 4D–G,J). Furthermore, collagen deposition, as assessed using Sirius Red staining, was markedly enhanced in the *Antxr2* ^iΔEC/iΔEC^ mice compared to that of controls, (Figures 4E,F). Analysis using hematoxylin and eosin (HE) staining revealed notable immune cell infiltration and significant changes in tissue structure, including the formation of pseudolobules in *Antxr2* ^iΔEC/iΔEC^ mice (Figure 4E). Hydroxyproline content in *Antxr2* ^iΔEC/iΔEC^ mice was significantly higher than that in the *Antxr2* ^loxP/loxP^ mice (Figure 4H). Serum samples were collected to assess various indicators of liver function damage. Mice in the knockout group showed higher levels of ALT, AST, and alkaline phosphatase (ALP) compared to those of the control group (Figure 4I). Based on these findings, mice lacking endothelial *Antxr2* showed more severe liver fibrosis after fibrosis modeling compared to controls.

### 3.5 ANTXR2 promoted MMP2 activation to degrade the ECM in human umbilical vein endothelial cells

**FIGURE 5:**
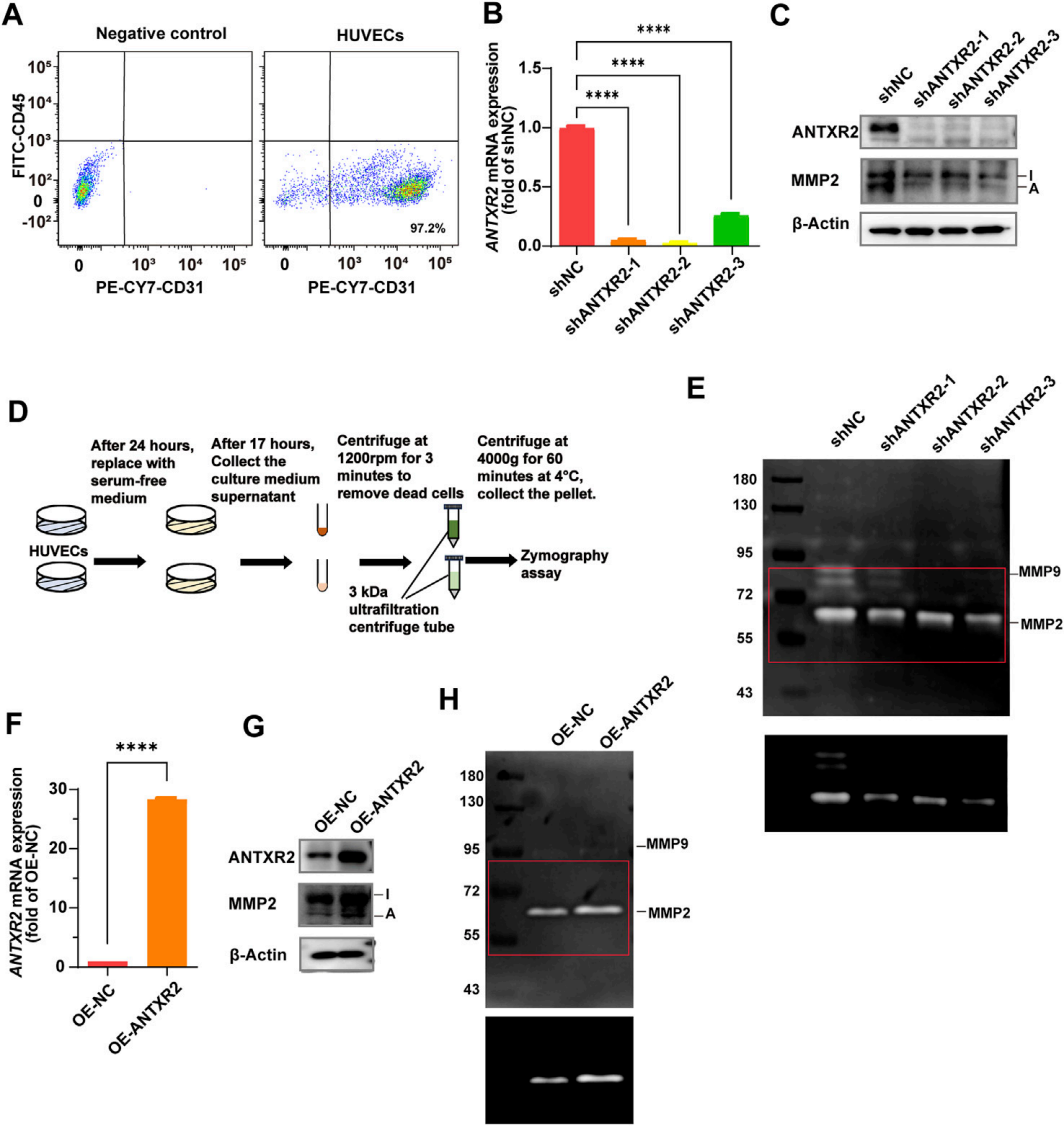
Anthrax toxin receptor 2 (ANTXR2) promotes matrix metalloproteinase 2 (MMP2) activation to degrade the extracellular matrix (ECM). **(A)** Dot plots showing the percentage of CD45^−^ CD31^+^ cell in primary human umbilical vein endothelial cells (HUVECs) as determined by flow cytometry. **(B, C)** Knockout efficiency of *ANTXR2* in HUVECs using short hairpin RNA (shRNA) (sh*ANTXR2*) as quantified using quantitative polymerase chain reaction (qPCR) and immunoblotting **(B, C)**. NC stands for negative control. Levels of activated MMP2 detected through western blot **(C)**. I stands for inactive MMP2 and A stands for active MMP2 (*n* = 3 per group). Statistical analysis of difference is performed with one-way ANOVA and Tukey’s test as *post hoc* analysis. Data are shown as mean ± S.D. *p* < 0.0001****. **(D)** Schematic of the zymography assay. First, after seeding the same number of HUVECs into a well plate, the cells are cultured for 24 h. Then, the serum-free medium is replaced and the cells are cultured for another 17 h before the supernatant is collected. The collected supernatant is centrifuged at 300 *g* for 3 min to remove cellular debris, and then it is concentrated using a 3 kDa ultrafiltration tube by centrifugation at 4,000 *g* for 60 min. Finally, the concentrated samples are used to detect MMP2 activity. **(E)** Zymography analysis of serum-free conditioned media from HUVECs in indicated group. The upper panel shows a low contrast image and is aimed to show the protein marker molecule size. The selected area of the red box was adjusted to high contrast and presented in the lower panel, in order to show the changes in activated MMP2 levels more clearly. The left column is labeled with protein marker molecule sizes (kDa). **(F,G)** Overexpression efficiency of *ANTXR2* in HUVECs (OE-*ANTXR2*), as quantified by qPCR and western blot **(F, G)**. Levels of activated MMP2 have been detected using the same methods **(G)**. (*n* = 3 per group). Student’s t-test is employed to determine significant differences. Data are expressed as mean ± S.D. *p* < 0.0001****. **(H)** Zymography analysis of serum-free conditioned media from HUVECs in OE-*ANTXR2* and controls. The upper panel shows low contrast and the bottom shows high contrast, as in Figure 5E. The left column is labeled with protein marker molecule sizes (kDa).

Reeves et al. found that ANTXR2 promotes the activation of MMP2 (C. V. Reeves et al., 2012). MMP2 is a collagenase that degrades collagen, gelatin, and other ECM components (Lu et al., 2011). To understand how ANTXR*2* exerted its antifibrotic effects in ECs, primary HUVECs were used to investigate the effect of ANTXR2 on MMP2 enzymatic activity. First, CD45^−^CD31^+^ cells accounted for 97.2% of primary HUVECs analyzed by flow cytometry (Figure 5A). Continuing with short hairpin RNA (shRNA) knockdown of *ANTXR2* expression in primary HUVECs (sh*ANTXR2*), both the mRNA and protein levels of ANTXR2 were suppressed (Figures 5B,C). Western blot analysis showed that when ANTXR2 was silenced in HUVECs, the levels of active MMP2 were significantly reduced (Figure 5C). Next, stable *ANTXR2* knockout and control HUVECs (shNC) were seeded at the same cell density in the culture plate and cultured for 24 h. After replacing the serum-free culture medium and incubating for an additional 17 h, the HUVEC supernatant was collected by centrifugation to remove dead cells. The concentrated culture medium was subjected to gelatin zymography to detect active MMP2 (Figure 5D). Compared to that of the shNC group, the sh *ANTXR2* group showed a significant decrease in active MMP2 (Figure 5E). Additional experiments were conducted to better define the activation of MMP2 by ANTXR2. HUVECs were transfected with a plasmid containing *ANTXR2* cDNA to induce its overexpression (OE-*ANTXR2*). The mRNA and protein levels of ANTXR2 were confirmed to be significantly higher than those in the control group (OE-NC) (Figures 5F,G). Overexpression of ANTXR2 in HUVECs significantly enhanced the formation of active MMP2 (Figure 5H). These data suggested that ANTXR2 degraded the ECM by increasing MMP2 activity.

### 3.6 *Antxr2* was specifically overexpressed in ECs by splenic injection of adeno-associated virus

**FIGURE 6:**
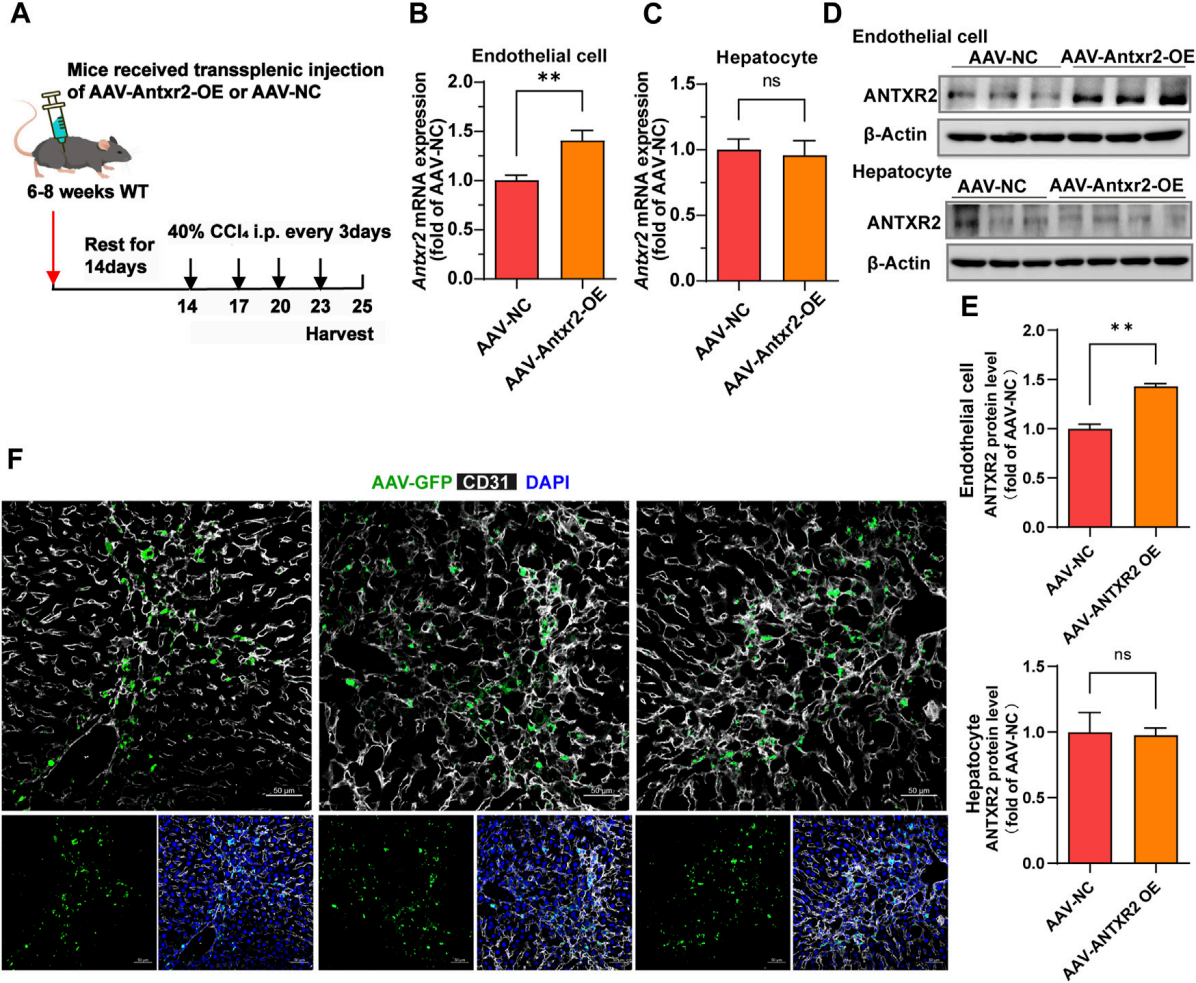
The endothelial-specific overexpression of anthrax toxin receptor 2 (ANTXR2) is induced by splenic injection of adeno-associated virus (AAV-Antxr2-OE). **(A)** Schematic of the treatment of endothelium-specific AAV-*Antxr2*-OE through trans-splenic injection. To induce selective overexpression of *Antxr2* in liver endothelial cells (ECs), AAV encoding EC-selective Tie1-driven *Antxr2* (AAV-*Antxr2*-OE) is injected into mouse liver via the spleen. After 21 days of AAV-Tie1-*Antxr2* injection, *Antxr2* is specifically overexpressed in the liver endothelium of mice. AAV-NC is the negative control, which is the blank control group. After 14 days of AAV injection, mice are subjected to 40% carbon tetrachloride (CCl_4_) through intraperitoneal injection every 3 days. Samples are harvested on the second day after the 4th consecutive CCl_4_ injection. **(B–E)** Endothelial-specific overexpression of Antxr2 was verified at the mRNA **(B, C)** and protein **(D)** levels by qPCR and western blot. Quantification of ANTXR2 protein level in endothelial cells and hepatocytes by densitometric analysis **(E)**. (*n* = 3–9 animals per group). Student’s t-test is employed to determine significant differences. Data are shown as mean ± S.D. **p < 0.01, ns = no significant difference. **(F)** AAV-green fluorescent protein (GFP, green) expression is co-stained with CD31 (white) in the liver sections from mice after AAV-*Antxr2*-OE or AAV-NC injection. Scale bar = 50 μm.

Because ANTXR2 promoted the activation of MMP2 to degrade the ECM, whether ANTXR2 overexpression might alleviate liver fibrosis was investigated. Disease treatment by AAV has been widely reported (D. Wang et al., 2019; Li and Samulski, 2020). Furthermore, AAV exhibited high expression levels starting from day 14 after injection, reaching a peak on day 21, and maintained high expression levels for approximately 3 months. An AAV vector with the Tie1 promoter (X. Zhang et al., 2022) was used to induce the expression of *Antxr2* in the endothelium (AAV-*Antxr2*-OE). AAV-NC served as a negative control. Multiple intraperitoneal injections of CCl_4_ cause liver injury, making it a rapid and effective model of liver fibrosis (Qing et al., 2022). Thus, a CCl_4_-induced fibrosis model was initiated in WT mice 14 days after the injection of endothelium-specifically overexpressing *Antxr2* AAV through the spleen, enabling *Antxr2* to be highly expressed in the endothelium after the third injection of CCl_4_ (Figure 6A). This allowed the determination of whether overexpressed *Antxr2* relieved the progression of fibrosis. To test whether AAV was specifically expressed in liver ECs, hepatocytes and liver CD45^−^CD31^+^ ECs were isolated to detect *Antxr2* expression. The AAV-*Antxr2*-OE group highly expressed *Antxr2* in the endothelium at both the mRNA and protein levels, but not in hepatocytes (Figures 6B–E). The plasmid vector of AAV-*Antxr2*-OE was also equipped with a green fluorescent protein (GFP) gene. Colocalization of GFP and the endothelial marker CD31 demonstrated that AAV-Antxr2-OE or AAV-NC indeed specifically targeted ECs (Figure 6F).

### 3.7 Specific overexpression of *Antxr2* in ECs greatly improved liver fibrosis

**FIGURE 7:**
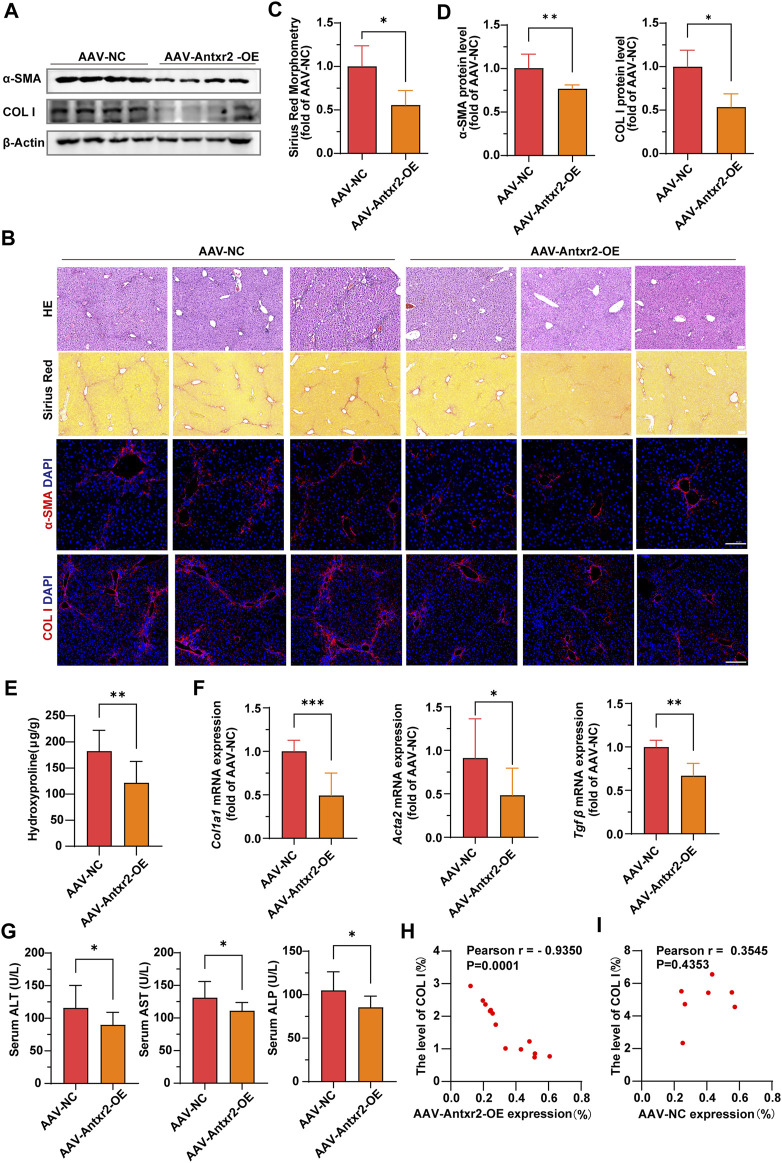
Specific overexpression of *Antxr2* in ECs by adeno-associated virus (AAV-*Antxr2*-OE) treatment significantly improved liver fibrosis **(A–D)** Wild-type (WT) mice are subjected to fibrosis modeling, which improves fibrosis after AAV-*Antxr2*-OE treatment. Levels of α-smooth muscle actin (α-SMA), collagen I **(A,B, D)**, and Sirius red staining **(B, C)** are measured to assess the degree of liver fibrosis. Hematoxylin and esosin (HE) staining **(B)** is used to observe the structure and morphology of tissues in described mice. Scale bar = 100 μm. (*n* = 4-6 animals per group). **(E)** Amounts of hydroxyproline in indicated mice (*n* = 8 animals per group). **(F)** Bar graph shows fibrosis-related genes mRNA expression level in AAV-NC group and AAV-*Antxr2*-OE group mice after fibrosis modeling (n = 6 animals per group). **(G)** Levels of serum alanine aminotransferase (ALT), aspartate aminotransferase (AST), and alkaline phosphatase (ALP) in indicated mice. (*n* = 7–9 animals per group). **(H, I)** A correlation analysis between the quantitative analysis of endothelial expression of *Antxr2* and collagen I in described mice (*n* = 7–13 animals per group). *p* < 0.05 is significant. R = −0.9350 indicates a strong negative correlation. Student’s t-test is employed to determine significant differences. Data are shown as mean ± S.D. *p* < 0.05*, *p* < 0.01**. *p* < 0.001***.

Analysis of α-SMA and collagen I expression, Sirius red staining and hydroxyproline content showed that liver fibrosis was significantly improved in the AAV-*Antxr2*-OE group compared with that of the control group after CCl_4_-induced liver fibrosis modeling (Figures 7A–F). Compared to the control group (AAV-NC), the AAV-*Antxr2*-OE group showed fewer pseudolobules and less inflammatory cell infiltration into the liver (Figure 7B). The serum levels of AST, ALT, and ALP in the AAV-*Antxr2*-OE group were significantly lower than those in the control group (Figure 7G). These data suggested that the endothelial-specific overexpression of *Antxr2* significantly suppressed the development of liver fibrosis and reduced liver damage. To better illustrate the relationship between endothelial-specific overexpression of *Antxr2* and the degree of fibrosis, a correlation analysis between the quantitative analysis of endothelial expression of *Antxr2* and collagen I was performed. As expected, the higher the expression efficiency of AAV-*Antxr2*-OE, the lower the collagen level, suggesting the more obvious the restoration of fibrosis. This is not observed in the AAV-NC controls (Figures 7H,I).

## 4 Discussion

Liver fibrosis, especially cirrhosis, is a major cause of morbidity and mortality in patients with chronic liver disease (Schuppan et al., 2018a). The early stages of advanced liver fibrosis in humans, including clinically compensated cirrhosis, may be reversed to a normal liver when the major triggers of chronic inflammation or injury are eliminated (Lee et al., 2015; Schuppan et al., 2018b). Therefore, their prevention or reversal has become the primary endpoint in clinical trials of novel liver-specific agents. Collagen is the most abundant ECM component involved in fibrosis. It increases up to 10-fold in cirrhosis and accounts for 50% of the dry weight (Schuppan, 1990), (D. Schuppan et al., 2001; Karsdal et al., 2017). In chronic diseases, such as liver fibrosis, regression depends on the collagenase activity of ECM-degrading MMPs (Kisseleva and Brenner, 2021).

**FIGURE 8:**
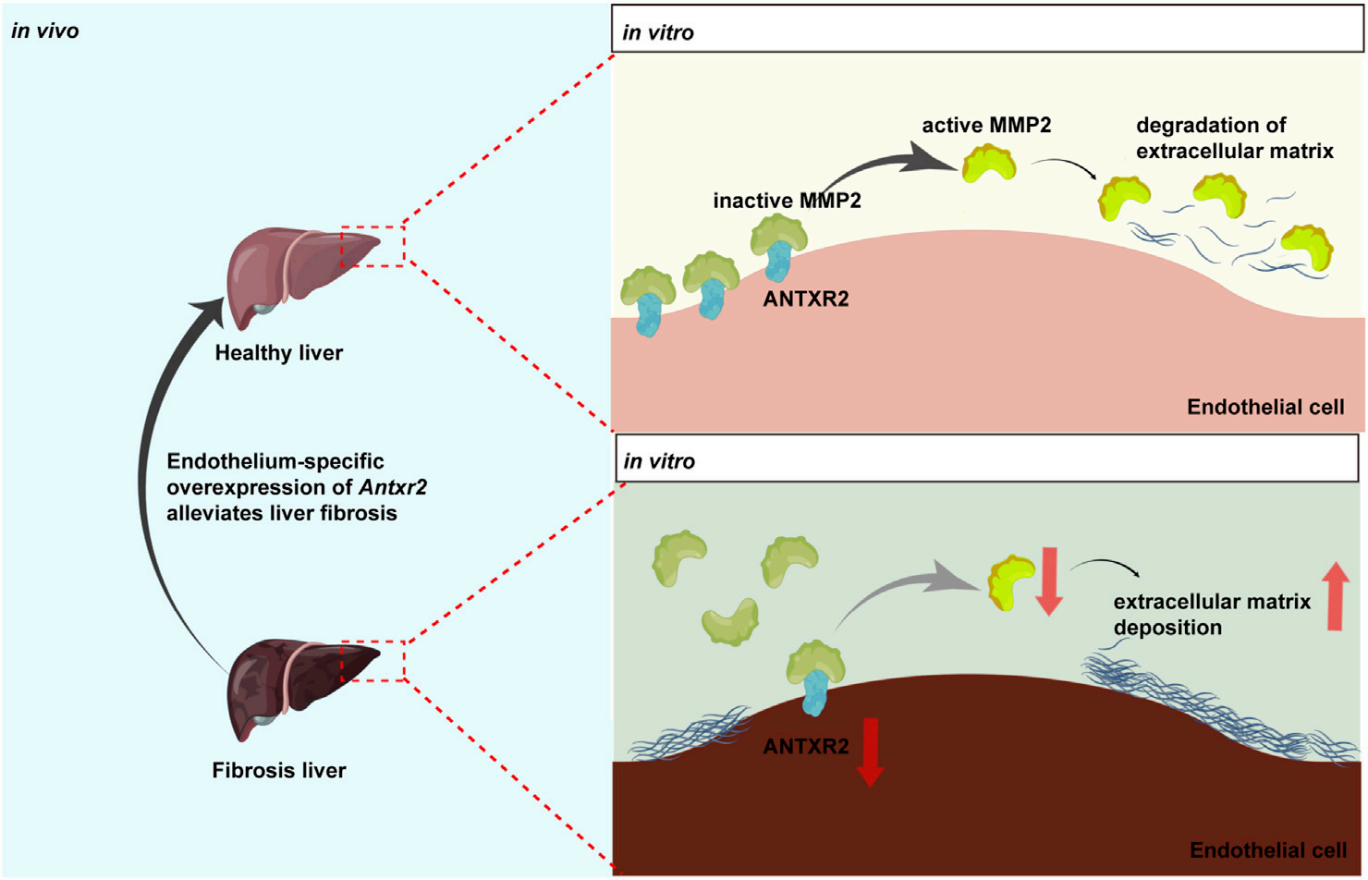
Anthrax toxin receptor 2 may function as a switch in liver fibrosis.Anthrax toxin receptor 2 (ANTXR2) expression in endothelial cells promotes the conversion of matrix metalloproteinase 2 (MMP2) from the zymogen form to the active form and degrades the extracellular matrix. Endothelium-specific overexpression of Antxr2 in liver after adeno-associated virus treatment may alleviate the development of liver fibrosis.

In this study, the transmembrane protein ANTXR2 in HUVECs promoted the conversion of MMP2 from its inactive form to its active form (Figure 8). Active MMP2 degrades collagen, which may promote ECM remodeling and fibrosis resolution (Bonnans et al., 2014; Zhou et al., 2018). However, the stimulatory effect of ANTXR2 on MMP2 activation may be indirect or mediated by MMP14. It has been reported that ANTXR2 forms a three-way complex with MMP14 and MMP2, promoting MMP2 activation and maintaining ECM homeostasis in the mouse reproductive tract (C. V. Reeves et al., 2012; C; Reeves et al., 2013). Future studies should explore the relationships between ANTXR2, MMP14, and MMP2 in ECs.

Active MMP2 levels are regulated by the expression of ANTXR2 in cells (C. Reeves et al., 2013). This is consistent with the current results, showing that the level of endothelial-specific overexpression of *Antxr2* was inversely correlated with the level of collagen deposition following AAV-*Antxr2*-OE injection via the spleen. Previous studies have shown that MMP2 specifically promotes ECM remodeling in the fibrotic liver (Onozuka et al., 2011; Radbill et al., 2011). However, it has not been elucidated whether anti-fibrotic role of hepatic endothelial ANTXR2 in the liver is dependent on the matrix-degrading function of MMP2.

Liver fibrosis increased in endothelium-specific Antxr2 knockout mice after fibrosis modeling. However it is not clear whether the above results are an inhibition of liver repair or an antifibrotic function, which may require liver repair modeling (Ding et al., 2014) to further explore both possibilities. The adult human body contains 10–60 trillion ECs (Augustin et al., 1994), which are indispensable cellular components of various organs. Whether the expression of ANTXR2 in the tissue-specific endothelium also affects the steady-state process of fibrogenesis and dissolution in other organs remains unclear.

Liver ECs are the main component of NPCs and have been reported to constitute approximately 5%–15% of NPCs(MacParland et al., 2018). However, when these two datasets were analyzed, the proportion of ECs in NPCs in some liver samples was very low, approximately 1%–3%, which might be caused by the method of tissue digestion, such as the enzymes used in the digestive system of the tissue, the time of digestion, or the degree of tissue fibrosis. Therefore, when using single-cell data to analyze changes in cell population proportions, it is necessary to include as many datasets as possible to increase the number of samples. Thus, other experimental methods, such as flow cytometry and tissue *in situ* immunostaining, were used to supplement the conclusions of the proportional change.

In summary, these data suggested that ANTXR2 functions as a switch in liver fibrosis. When *Antxr2* expression in liver ECs was decreased, liver repair may be inhibited, resulting in the aggravation of fibrosis, whereas increased *Antxr2* expression in liver ECs after AAV treatment effectively improved liver fibrosis and restored normal liver function (Figure 8).

## Data Availability

Publicly available datasets were analyzed in this study. This data can be found here: https://www.ncbi.nlm.nih.gov/geo/query/acc.cgi?acc=GSE136103; https://www.ncbi.nlm.nih.gov/geo/query/acc.cgi?acc=GSE181483.
