# Supplementary material for: Endothelial anthrax toxin receptor 2 plays a protective role in liver fibrosis
Source: Front Cell Dev Biol. 2024 Jan 23;11:1278968. doi: 10.3389/fcell.2023.1278968 (PMC10844529; doi:10.3389/fcell.2023.1278968)
Supplement: Supplementary file 1 [file Table1.docx]

Supplementary Material

Hepatic endothelial expression of ANTXR2 promotes MMP2 activation and improves liver fibrosis

Xiaojuan Huang^1†^, Liyin Zhang^1†^, Wei Luo^1†^, Yu Zeng^1^, Xiaoxue Li^1^, Nan Yang^1^, Wenwen Huang^1^, and Bi-Sen Ding^1*^

*** Correspondence:** Bi-Sen Ding*: [dingbisen@scu.edu.cn](mailto:dingbisen@scu.edu.cn)

# Table 1

| M-genotyping VE CAD Cre | F：GCC TGC ATT ACC GGT CGA TGC AAC GA |
| --- | --- |
|  | R：GTG GCA GAT GGC GCG GCA ACA CCA TT |
| M-genotyping ANTXR2 | F：CAGAACTCTAGGTCAGGGGC |
|  | R：CTTATGCCTCATCCCTCCGC |
| M-qPCR Antxr2 | F：TCAGTCACGATGGCAGTGTC |
|  | R：GCTCACTGGCTTCTCACTCTT |
| M- qPCR β-Actin | F：GCTGTATTCCCCTCCATCGTG |
|  | R：CACGGTTGGCCTTAGGGTTCAG |
| M-qPCR-col1a1 | F: GTCCCTGAAGTCAGCTGCATA |
|  | R: TGGGACAGTCCAGTTCTTCAT |
| M-qPCR-Acta2 | F: AGCCATCTTTCATTGGGATGG |
|  | R: CCCCTGACAGGACGTTGTTA |
| M-qPCR-Tgf β | F: TGACGTCACTGGAGTTGTACGG |
|  | R: GGTTCATGTCATGGATGGTGC |
| shRNA targeting sequence: Antxr2 | |
| shAntxr2-1 | F:CCGGTGCTCAGTCATGTACTGAAATCTTCAAGAGAGATTTCA  GTACATGACTGAGCTTTTTTG |
|  | R:AATTCAAAAAAGCTCAGTCATGTACTGAAATCTCTCTTGAAG  ATTTCAGTACATGACTGAGCA |
| shAntxr2-2 | F:CCGGTGCAGTGTTCTCTGCACTTACATTCAAGAGATGTAAGTG  CAGAGAACACTGCTTTTTTG |
|  | R:AATTCAAAAAAGCAGTGTTCTCTGCACTTACATCTCTTGAATG  TAAGTGCAGAGAACACTGCA |
| shAntxr2-3 | F:CCGGTGGAGATGAGGTTTGTATATGGTTCAAGAGACCATATAC  AAACCTCATCTCCTTTTTTG |
|  | R:AATTCAAAAAAGCGGATTTGACCTGTACTTCATCTCTTGAAT  GAAGTACAGGTCAAATCCGCA |
| Overexpression Antxr2 | |
| OE-Antxr2 | F:CGACTCTAGAGGATCCATGGTGGCGGAGCGG |
|  | R:CGAGAAGCTTGTCGACAGCAGTTAGCTCTTTCTCAATACATTCC |

**2 Supplementary figures 1-2**


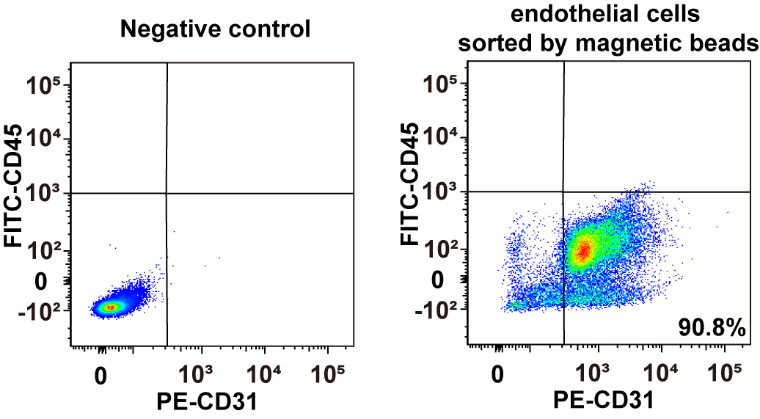


**Supplementary figure 1. The vast majority of cells sorted by magnetic beads are endothelial cells.**

Dot plots show the percentage of CD45^-^CD31^+^ cell in cells sorted by magnetic beads as determined by flow cytometry.


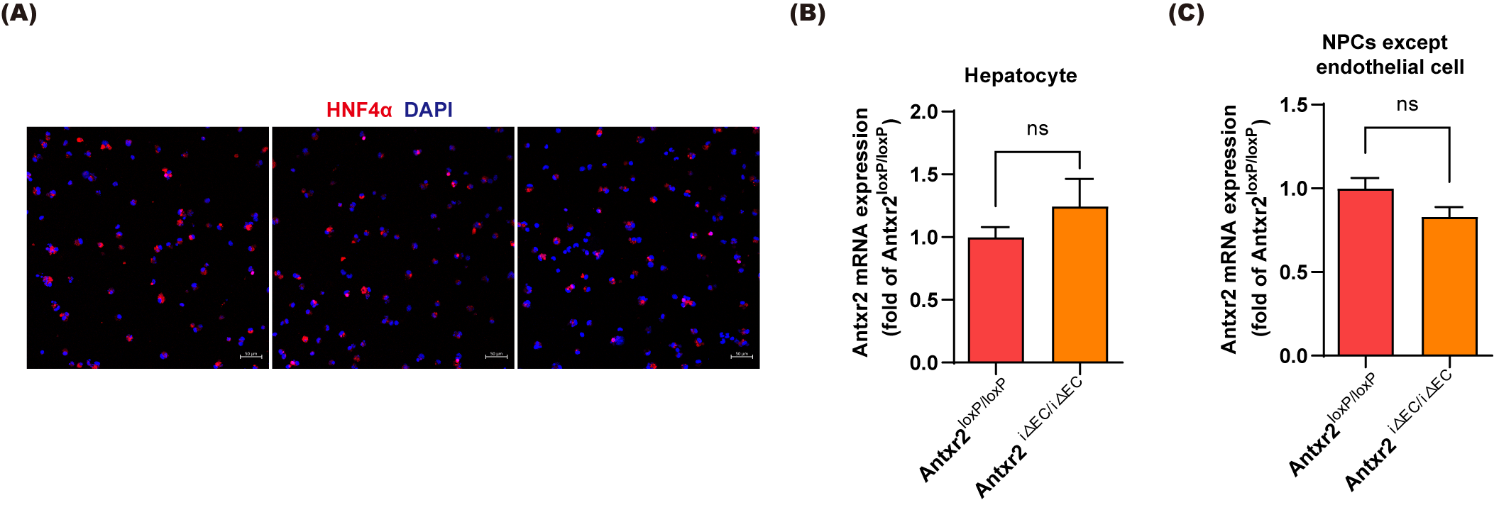


**Supplementary figure 2. No Significant difference in Antxr2 mRNA Expression Level in Liver Cells Excluding Endothelial Cells between *Antxr2* ^iΔEC/iΔEC^ and *Antxr2*^loxP/ loxP^ mice**

1. Primary hepatocytes were stained with HNF4α as a hepatocyte marker molecule. Immunofluorescence shows the purity of hepatocytes.
2. Antxr2 mRNA expression in hepatocytes and NPCs except endothelial cells from *Antxr2* ^iΔEC/iΔEC^ and *Antxr2*^loxP/ loxP^ mice. (n = 6–9 animals per group). Student’s t-test is employed to determine significant differences. Data are shown as mean ± S.D. ns = no significant difference.
